# Seroprevalence of *Toxoplasma gondii* and associated risk factors for infection in the Netherlands: third cross-sectional national study

**DOI:** 10.1017/S095026882300122X

**Published:** 2023-07-28

**Authors:** Oda E. van den Berg, Kamelia R. Stanoeva, Rens Zonneveld, Denise Hoek-van Deursen, Fiona R. van der Klis, Jan van de Kassteele, Eelco Franz, Marieke Opsteegh, Ingrid H. M. Friesema, Laetitia M. Kortbeek

**Affiliations:** 1 Centre for Infectious Disease Control, National Institute for Public Health and the Environment (RIVM), Bilthoven, The Netherlands; 2 European Public Health Microbiology Training Programme (EUPHEM), European Centre for Disease Prevention and Control (ECDC), Stockholm, Sweden; 3 Department of Medical Microbiology and Infection Prevention, Academic Medical Centre, Amsterdam University Medical Centre, Amsterdam, The Netherlands

**Keywords:** risk factors, serology, seroprevalence, The Netherlands, *Toxoplasma gondii*

## Abstract

A third nationally representative serosurvey was performed to study the changes in *Toxoplasma gondii (T. gondii)* seroprevalence in the Netherlands over a 20-year time span and to identify and confirm risk factors for acquired toxoplasmosis. This cross-sectional study (conducted in 2016/2017) was designed similarly to the previous two studies (1995/1996 and 2006/2007) and included a questionnaire and serum sampling among Dutch residents. Factors associated with seropositivity for *T. gondii* were determined using multivariable analysis of the questionnaire-derived data. The earlier observed decrease in *T. gondii* seroprevalence between 1995/1996 and 2006/2007 (from 40.5% to 26.0%) did not continue into 2016/2017 (29.9%). Similarly to the previous studies, the seroprevalence increased with age and varied among regions. In all studies, higher *T. gondii* seropositivity was associated with increasing age, lower educational level, not living in the Southeast, and eating raw or semi-cooked pork. The incidence of congenital toxoplasmosis was estimated at 1.3/1000 (95% CI 0.9–1.8) live-born children in 2017. As the seroprevalence of *T. gondii* in the Netherlands did not decrease over the last decade, an increase in public health awareness is needed and prevention measures may need to be taken to achieve a further reduction in *T. gondii* infections in the Netherlands.

## Introduction

The protozoan *Toxoplasma gondii (T. gondii)* is a zoonotic and ubiquitous parasite infecting more than one-third of the global human population [1]. Warm-blooded animals are hosts, but only Felidae, such as domestic cats, are definitive hosts and shed oocysts into the environment. Human infection occurs through the consumption of tissue cysts in undercooked meat from livestock, through the ingestion of sporulated oocysts from contaminated water or food, and, much less frequently, through the transplantation of organs with tissue cysts [2]. When immunocompetent people become infected, the disease is most typically asymptomatic or mild with flu-like symptoms [2]. There is a small risk of developing ocular toxoplasmosis, which can lead to blindness [3].

*T. gondii* infection may have severe consequences for two main risk groups: pregnant women and immunocompromised individuals [3]. Transplacental transmission of *T. gondii* may result in spontaneous abortion, prematurity, stillbirth, or neurological or ocular damage to the foetus [4]. Eye lesions from congenital infection are often not identified at birth but occur in 20–80% of congenitally infected persons by adulthood [3]. In immunocompromised people, toxoplasmosis almost always happens as a result of the reactivation of latent infection and can result in neurological symptoms, including headache, disorientation, drowsiness, hemiparesis, reflex changes, and convulsions [5]. Acute acquired *T. gondii* infection in immunocompromised patients may also involve encephalitis, pneumonia, retinochoroiditis, and other disseminated systemic diseases [6].

Risk factors for detectable *T. gondii* antibodies were described in two earlier Dutch serosurveys and included increasing age, urban living, low educational level, cat ownership, raw meat consumption, and gardening [7, 8]. Exactly how these risk factors relate to different routes of transmission remains under debate. The association between *T. gondii* seroprevalence and cat ownership indicates the relevance of the oocyst-based transmission route in human infection [9]. However, the association of the consumption of raw meat with human infection indicates a relevant tissue cyst-based transmission route [10].

The estimated incidence of congenital toxoplasmosis (CT) and overall toxoplasmosis-related disability-adjusted life years (DALYs) in the Netherlands are relatively high, which warrants continuous evaluation of seroprevalence and risk factors [11]. Many European countries have reported various degrees of decrease in seroprevalence between 1980 and recent years, either in the general population or in women of reproductive age [12, 13]. Here, we present data from the third national serosurvey collected in 2016–2017. These data will help monitor age-related seroprevalence dynamics in a 20-year time span, confirm and identify risk factors, and help determine relevant public health interventions.

## Material and methods

### Study population and questionnaire

The serosurvey, known as PIENTER-3 or P3, was originally conducted by the National Institute for Public Health and the Environment (RIVM) to evaluate the (long-term) epidemiological effects of the nationwide immunisation programme. The acronym PIENTER stands for ‘Peiling immunisatie effect Nederland ter evaluatie van het Rijksvaccinatieprogramma’. This survey follows a similar design to the two previous national serosurveys, PIENTER-1 (P1) in 1995/1996 and PIENTER-2 (P2) in 2006/2007, which are described elsewhere [14–17]. Kortbeek et al. [7] published *T. gondii* seroprevalence results for P1 and Hofhuis et al. [8] for P2. In short, a two-stage cluster sampling technique was used to draw a sample of Dutch residents aged 0–89 years. Cluster sampling was done to ensure that the included municipalities represented all regions in the Netherlands and all age groups. Forty municipalities were sampled within five regions proportional to size, including an oversampling of people living in low vaccination coverage (LVC) areas and non-Western migrants living in the Netherlands. To estimate seroprevalences, people living in LVC areas were excluded from the national sample (NS). This was done because the NS (including the oversampling of non-Western migrants) is rather comparable to the Dutch population, especially regarding urbanisation degree and religion [17]. As life expectancy is increasing, the maximum age in this study was extended from 79 (in the previous surveys) to 89 years. Sampling was performed independently for P1 to P3. Therefore, the overlap between selected municipalities was only coincidental. The data collection took place from 1 February 2016 to 16 October 2017 and included a questionnaire and serum sampling.

The study proposal was approved by the Medical Ethics Committee Noord-Holland (METC number: M015–022), written informed consent was obtained from all adult participants, and parents or legal guardians of minors were included in the study.

### Antibody assay

The same methods for antigen production using *T. gondii* strain and the same batch of freeze-dried control sera were used as in the previous two national serosurveys [7, 8]. Therefore, data from all PIENTER studies can be compared.

After collection, the sera were stored at −20˚C until testing. Sera were diluted 1:20 and tested in duplicate for IgG antibodies to *T. gondii* in a sandwich ELISA [18]. The antigen was derived from a crude extract of a *Toxoplasma* RH strain, and the conjugate was a peroxidase-labelled anti-human IgG conjugate (Dako, Denmark). A ratio ≥ 1.0 between the optical density (OD) of the samples and the mean OD of the cut-off controls was considered positive. Samples with discrepant results for duplicates (i.e. one above and one below the cut-off value) were excluded.

### Statistical analysis

To determine the seroprevalence of *T. gondii* IgG antibodies representative of the general population of the Netherlands, seroprevalence was weighted within each municipality for age and gender and also for ethnicity and urbanisation degree, up to their proportion in the total population of the Netherlands (on 1 January 2017) [19]. Seroprevalence analyses were further adjusted for the two-stage cluster sampling by considering the strata (i.e. five regions) and clusters (i.e. 40 municipalities).

To estimate the incidence of live-born children with CT, the number of live-born children in the Netherlands in 2017 per age group of the mother was multiplied by the age-specific force of infection among the susceptible population of women of reproductive age. The mother-to-child transmission per trimester was based on previous studies [20]. The force of infection (FOI) was estimated as the derivative of the age-specific seroprevalence relative to one minus the age-specific seroprevalence [21]. The age-specific seroprevalence was estimated by survey-weighted logistic regression using natural cubic splines with three degrees of freedom. Because of the non-linear relation between prevalence and FOI, the uncertainty in the FOI estimates was calculated using 1,000 Monte Carlo samples from the estimated prevalence. Logistic regression analysis was used to determine whether any of the selected variables were independent predictors of seropositivity for *T. gondii*, after adjustment for age group and gender. In contrast to the weighted seroprevalence estimation, all 5,878 participants were included in the logistic regression to increase power, as living in a low immunisation coverage municipality was not significantly associated with *T. gondii* seropositivity (*P*-value = 0.154) or with any of the relevant demographics. Variables that reached a significance level of *P*-value <0.10 in the univariable analyses for the 0–19 and > 20 years of age groups were selected for inclusion in the multivariable logistic regression model. Odds ratios (ORs) were corrected for gender and age. Models for the 0–19 and > 20 years of age groups were developed using multivariable logistic regression with backwards elimination. A two-sided p-value of ≤0.05 was considered statistically significant. Multicollinearity was tested using a correlation matrix and the variance inflation factor. All statistical analyses were performed with R [22].

## Results

### Prevalence of Toxoplasma gondii IgG antibodies

**Figure 1.**
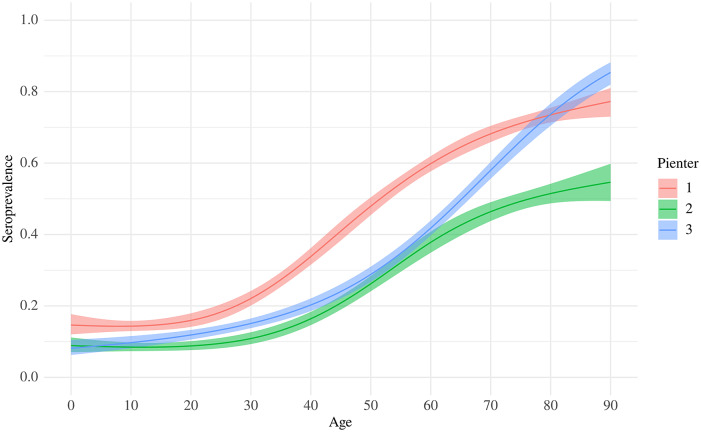
Estimated age-specific weighted prevalence of *Toxoplasma gondii* IgG antibodies in the three Dutch PIENTER serosurveys.

**Figure 2.**
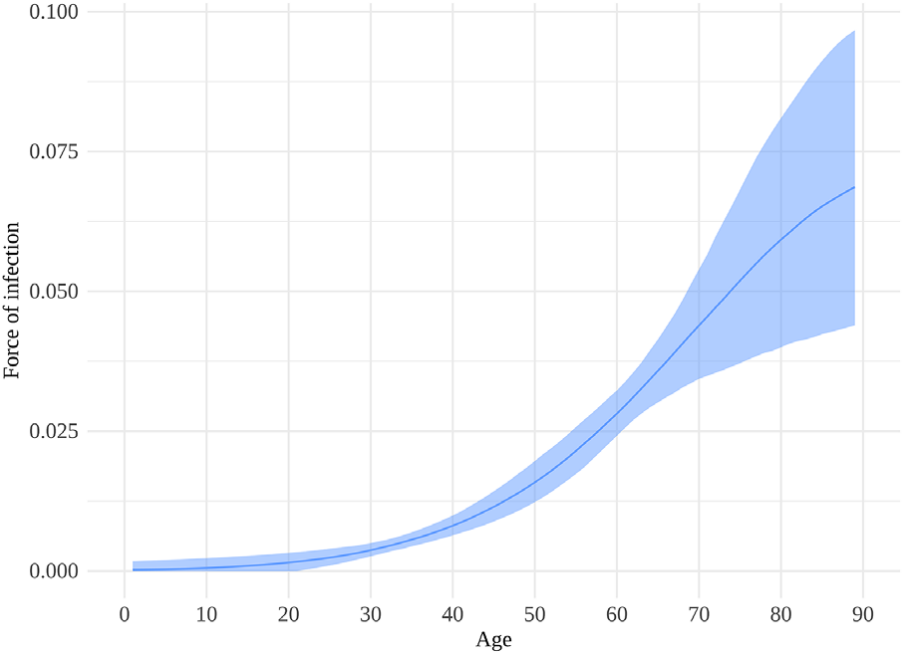
Estimated age-specific force of infection for Toxoplasma gondii in women (P3).

The overall weighted estimate of *T. gondii* seroprevalence for people aged 0–89 years was 30.9% (95% CI 29.4–32.4). The average seroprevalence for children and adolescents aged 0–19 years was 10.9% (95% CI 9.12–12.7) and that for adults aged 20–89 years was 36.7% (95% CI 34.7–38.6). The average seroprevalence for women of reproductive age (15–49 years) was 17.5% (95% CI 15.2–20.1). No differences were found between men and women (31% versus 28%, *P*-value = 0.18). As can be seen in Figure 1, seroprevalence increased with age, with the steepest slope in people aged 60 to 70 years, resulting in seroprevalences of more than 80% among the highest age groups. The highest seroprevalences were seen in the Northwest and the lowest in the Southeast. The group of 0–19 years old (*n* = 989) showed a higher seroprevalence in the Northwest and the Northeast compared with the Southeast (8%, OR: 2.05 resp. 2.11, *P*-value = 0.03 resp. 0.02). Figure 2 shows the age-specific FOI in women in P3, which was used to estimate the incidence of congenital toxoplasmosis. Based on the FOI in women of reproductive age and the likelihood of transmission per trimester from mother to child based on previous studies [20], the incidence of congenital toxoplasmosis was estimated at 1.3/1000 (95% CI 0.9–1.8) live-born children in 2017.

### Predictors of seropositivity for T. gondii IgG antibodies

Univariable analyses were performed on all selected variables with a priori adjustment for age and gender (Supplementary Table S1). Variables that were associated with seropositivity in multivariable analyses are shown in Table 1. For those <20 years of age, age and living in the Northeast or Northwest of the Netherlands were independently associated with *T. gondii* seropositivity. For those aged 20–89 years, independently associated variables included age, not living in the Southeast of the Netherlands, being born outside of the Netherlands, low educational level, eating meat, having kept cattle for the past 5 years, eating raw pork, and having ever been abroad to Asia, Africa, or America. The risk factors associated with seropositivity in participants aged 0–19 and 20–89 years are shown in Table 1.

**Table 1.** Uncorrected prevalence of specific antibodies to *Toxoplasma gondii* (%) and multivariable logistic regression analyses of risk factors associated with seropositivity in participants aged 0–19 and 20–89 years

	Age group of 0–19 years (*n* = 1,250)				Age group of 20–89 years (*n* = 4,625)			
	*N*	Prevalence IgG %	Multivariable OR^a^ (95% CI)	*P*-value	*N*	Prevalence IgG %	Multivariable OR^a^ (95% CI)	*P*-value
Geographical region^b^								
Southeast	207	8%	1.0		968	26%	1.0	
Central	342	11%	1.4 (0.8–2.6)	0.252	1,050	34%	1.6 (1.3–2.0)	<0.001
Northwest	212	15%	1.9 (1.0–3.6)	0.046	717	43%	2.0 (1.6–2.5)	<0.001
Northeast	277	16%	2.0 (1.1–3.8)	0.028	891	36%	1.6 (1.3–2.0)	<0.001
Southwest	212	6%	0.8 (0.4 1.6)	0.502	999	32%	1.7 (1.4–2.1)	<0.001
Country of birth								
The Netherlands	1,178	11%	ns		4,027	32%	1.0	
Other	72	15%			595	44%	1.5 (1.2–1.8)	<0.001
Educational level^c^								
High	350	9%	ns		1,527	29%	1.0	
Medium	436	12%			1,549	26%	1.0 (0.8–1.2)	0.711
Low	399	11%			1,281	49%	1.3 (1.1–1.6)	0.001
Eating meat								
No	22	23%	ns		102	18%	1.0	
Yes	1,140	11%			4,260	33%	2.1 (1.2–3.8)	0.008
missing	88	13%			263	42%	3.1 (1.7–5.9)	<0.001
Kept cattle during the past 5 years								
No	1,228	11%	ns		4,578	33%	1.0	
Yes	19	26%			43	44%	2.8 (1.4–5.5)	0.003
Eating raw pork								
No	867	11%	ns		2,886	32%	1.0	
<=3 days a month	137	9%			812	32%	1.3 (1.0–1.5)	0.014
>1 day a week	19	21%			100	34%	1.5 (0.9–2.4)	0.084
missing	227	13%			827	41%	1.5 (1.3–1.8)	<0.001
Having ever been abroad to Asia, Africa, or America								
No	867	10%	ns		2,159	31%	1.0	
Yes	380	12%			2,462	36%	1.2 (1.0–1.3)	0.0227

Abbreviations: 95% CI, confidence interval; OR, odds ratio; ns, not significant.

a Adjusted for age and gender.

b The following categories were used for the educational level of those aged ≥15 years and of parents for those aged <15 years: ‘low’ (primary school, lower vocational or lower general secondary education), ‘medium’ (intermediate vocational or intermediate general secondary and higher general secondary education), and ‘high’ (higher vocational secondary education and university education).

c The geographical regions were based on the Dutch provinces: ‘Central’ (Utrecht and Gelderland), ‘Southeast’ (Noord-Brabant and Limburg), ‘Northwest’ (Noord-Holland and Flevoland), ‘Southwest’ (Zeeland and Zuid-Holland), and ‘Northeast’ (Groningen, Drenthe, Overijssel, and Friesland).

### Comparison with previous national surveys

Table 2 displays the prevalence of *T. gondii* antibodies in 2006/2007 and 2016/2017, categorised by gender, degree of urbanisation, being born in the Netherlands, and ethnicity. For people aged 0–79 years in the Netherlands, the overall weighted estimate of *T. gondii* seroprevalence decreased from 40.5% (95% CI 37.5–43.4) in 1995/1996 to 26.0% (95% CI 24.0–28.0) in 2006/2007 and increased to 29.9% (95% CI 28.3–31.4) in 2016/2017. Including participants aged 80–89 years in 2016/2017 did not significantly change the overall weighted seroprevalence (30.9%; 95% CI 29.4–32.4). The average seroprevalence for women of reproductive age (15–49 years) decreased from 35.2% (95% CI 32.9–38.6) in 1995/1996 to 18.5% (95% CI 16.2–20.7) in 2006/2007, but remained stable in 2016/2017 (17.5%; 95% CI 15.2–20.1). The incidence of congenital toxoplasmosis shows a similar pattern and was estimated at 3.4/1000 (95% CI 2.8–3.9) live-born children in 1996, 1.5/1000 (95% CI 1.1–2.0) in 2007, and 1.3/1000 (95% CI 0.9–1.8) in 2017. Among people aged 65 years and older, there was no significant difference in seroprevalence between 1996/1997 and 2016/2017. The seroprevalence in the age group of 75–79 years was significantly higher in 2016/2017 compared with 2006/2007 (Figure 1).

**Table 2.** Prevalence^a^ of *Toxoplasma gondii* antibodies in 2006/2007 and 2016/2017, stratified for gender, degree of urbanisation, being born in the Netherlands, and ethnicity

		2006/2007 (*n* = 5,541)			2016/2017 (*n* = 4,759)		
			Prevalence IgG			Prevalence IgG	
		*n*	%	(95% CI)	*n*	%	(95% CI)
Overall		5,541	26.0%	(24.0–28.0)	4,759	30.9%	(29.4–32.4)
Male		2,522	25.9%	(23.2–28.5)	2,147	31.4%	(28.8–34.2)
Female		3,019	26.1%	(24.0–28.2)	2,621	30.3%	(28.3–32.5)
Female of reproductive age (15–49)		1,195	18.5%	(16.2–20.7)	1,339	17.3%	(14.8–20.0)
Urbanisation^b^							
Rural area		1,861	24.9%	(21.6–28.3)	2,178	28.7%	(25.5–32.2)
Urban area		3,682	26.5%	(23.9–29.2)	2,581	32.6%	(29.8–35.5)
Country of birth							
The Netherlands		4,681	26.3%	(24.4–28.1)	4,116	29.8%	(27.9–31.7)
Other		801	24.5%	(19.8–29.1)	643	38.5%	(32.6–44.7)
Ethnicity							
Autochthonous		4,154	27.0%	(25.0–29.1)	3,758	30.6%	(28.7–32.6)
Immigrant from Western country		386	25.9%	(22.0–29.9)	322	32.4%	(27.1–38.2)
Immigrant from Morocco/Turkey		328	19.4%	(14.4–24.3)	112	33.3%	(22.6–45.4)
Immigrant from Surinam/Aruba/Antilles		336	19.3%	(14.5–23.7)	235	30.0%	(24.0–36.6)
Immigrant from other non-Western countries		339	18.8%	(15.0–22.5)	332	30.7%	(25.5–36.3)

CI, confidence interval.

a Weighted prevalence for age, gender, ethnicity, and urbanisation degree.

b The following categories were used for the level of urbanisation: ‘urban’ (>1,500 addresses/km^2^) and ‘rural’ (<1,500 addresses/km^2^).

For those <20 years of age, ‘living in the Northeast’ was protective in P1, but was associated with an increased risk in P3. Significant predictors in this age group also included ‘country of birth’, ‘urbanisation degree’, and ‘gardening’ in P1 and ‘having kept sheep or cattle in the past 5 years’ in P2. None of these variables were significant predictors in P3.

For those 20–79 years of age, ‘low educational level’ and ‘living outside of the Southeast of the Netherlands’ were significant predictors in all three surveys. ‘Keeping a cat in the past 5 years’ was a significant predictor in P1 and P2, but not in P3. ‘Eating meat’ was only a significant predictor in P3 and ‘consumption of raw or undercooked pork’ was a significant predictor in P2 and P3, but not in P1. A comparison of the associated risk factors for those aged 20–79 years is shown in Table 3. Extending the maximum age to 89 years in P3 resulted in the same risk factors.

**Table 3.** Comparison of the associated risk factors for those aged 20-79 years between the different PIENTER studies

	1995/1996 (*n* = 7,521)	2006/2007 (*n* = 7,030)	2016/2017 (*n* = 5,875)
	Odds ratio	Odds ratio	Odds ratio
Marital status			
Married/living together/unmarried	1.0	n.s.	N/A
Divorced/widowed	1.4 (1.1–1.7)		
Educational level			
High	1.0	1.0	1.0
Medium			1.0 (0.8–1.1)
Low	1.4 (1.2–1.7)	1.4 (1.2–1.7)	1.3 (1.1–1.5)
Region			
Southeast	1.0	1.0	1.0
Central	1.8 (1.5–2.2)	2.0 (1.5–2.5)	1.7 (1.4–2.1)
Northwest	3.1 (2.5–3.8)	2.3 (1.8–3.0)	1.9 (1.5–2.4)
Northeast	1.8 (1.5–2.1)	1.5 (1.2–1.9)	1.7 (1.3–2.1)
Southwest	1.9 (1.6–2.3)	1.9 (1.5–2.3)	1.6 (1.3–2.0)
Urbanisation^a^			
Very high	sign	sign	n.s.
Keeping a cat in the past 5 years			
No	1.0	1.0	n.s.
Yes	1.2 (1.1–1.4)	1.4 (1.2–1.6)	
Consumption of raw or undercooked pork			
No	n.s.	1.0	1.0
Yes		1.4 (1.1–1.6)	1.3 (1.1–1.6)
Missing			1.5 (1.2–1.8)
Country of birth			
The Netherlands	n.s.	n.s.	1.0
Other			1.3 (1.1–1.7)
Eating meat			
No	n.s.	n.s.	1.0
Yes			2.7 (1.5–5.0)
Missing			2.8 (1.6–5.4)
Having kept cattle for the past 5 years			
No	n.s.	n.s.	1.0
Yes			3.1 (1.5–6.1)
Having ever been abroad to Asia, Africa, or America			
No	n.s.	n.s.	1.0
Yes			1.2 (1.0–1.3)

Abbreviations: N/A, not included in the questionnaire; n.s., not significant; sign, significant.

a Urbanisation degree was categorised in different ways for the different studies and ORs could therefore not be compared.

## Discussion

Interestingly, the strong decrease in *T. gondii* seroprevalence between 1996/1997 (40.5%) and 2006/2007 (26.0%) did not continue into 2016/2017 (29.9% for 0–79 years of age). The seroprevalence in women of reproductive age in 2016/2017 (17.5%) was lower than in 1995/1996 (35.2%; 95% CI 32.9–38.6), but not different from 2006/2007 (18.5%; 95% CI 16.2–20.7). This was surprising, since other high-income countries have been reporting decreasing *T. gondii* seroprevalences. A national perinatal survey in France, conducted in 1995, 2003, 2010, and 2016, showed a continuously decreasing seroprevalence of *T. gondii* infection among pregnant women from 54% in 1995 to 31% in 2016 [13]. In addition, a decreasing seroprevalence has been reported in a longitudinal study among immunocompromised patients in Paris [23]. However, due to differences in the study populations and varying consumption habits and soil exposure between countries in general, the relative importance of sources of infection could differ greatly, leading to different trends. Moreover, the seroprevalence in pregnant women in France was much higher to begin with and it is possible that the seroprevalence will plateau at a lower level. In Portugal, a study of the general population showed an overall decrease from 47% in 1979/1980 to 36% in 2002/2003 to 22% in 2013 [24]. However, the difference in time points makes it difficult to compare our data. Based on the age-related increase in seroprevalence in women of reproductive age, the estimated incidence of congenital toxoplasmosis in our study (1.3/1,000 (0.9–1.8) in live-born children in 2017) was relatively high compared with other European countries [13, 25]. However, as women are advised to avoid potential sources of *T. gondii* infection during pregnancy, our method could overestimate the actual incidence of congenital toxoplasmosis.

As noted in the previous national serosurvey studies, seropositivity increased with increasing age. However, until 60 years of age the curve of P3 is more or less comparable to P2, but from 60 years of age and older, there is a substantial increase in seroprevalence and the curve starts moving towards the P1 curve. This may indicate a strong FOI in this age group. However, since the infection is presumed to persist lifelong, this could alternatively be explained by a birth cohort effect. In 1995/1996, the steepest slope was observed in the age group of 25–44 years [7]. This does, 20 years later, partly concern the same birth cohort.

In further agreement with the previous serosurvey studies, seroprevalence was higher in those who had lower education and those who lived in more urbanised areas. In addition, people living in the Southeast of the Netherlands have consistently had a lower seroprevalence compared with the rest of the country since 1995, which may, for example, be due to dietary habits or a lower level of oocyst contamination in the environment. The inconsistencies in the geographical regions in the younger age group are likely due to the small number of cases.

Transmission of *T. gondii* infection is subject to complex environmental, socioeconomic, and dietary factors and can change over time. In general, the observed decreases in seroprevalences in other countries have partly been attributed to reduced exposure to contaminated meat due to confined husbandry, changes in food storage and preparation, and changing dietary habits [24, 26]. Others have speculated on the influence of changes in attitudes towards cat ownership on environmental contamination [26, 27].

Within the Netherlands, having kept a cat in the past 5 years was a risk factor in P1 and P2, but not in P3. In addition, the consumption of raw or poorly cooked pork was a risk factor in P2 and P3, but not in P1, and eating meat in general was a risk factor only in P3. This could possibly be due to changes in soil or cat faeces-related hygiene and food safety behaviours, indicating a shift from oocyst-based transmission routes to more tissue cyst-based transmissions as the main contributors to human infection. However, this could also be due to a misclassification bias, since infection in seropositive individuals may have happened long before their exposure to risk factors.

Since cats are the only known definitive hosts for *T. gondii*, they are essential to the persistence of *T. gondii* in hosts. In the Netherlands, about 24% of the households owned a cat in 2017 [28] and the overall population of owned cats in the Netherlands was estimated at 3.1 million in 2015 and 2.6 million in 2017 [29]. However, due to cats’ fastidious grooming habits, the short duration of oocyst shedding, and the fact that when freshly passed in stools, the oocysts are not infective, and direct contact with cats is not thought to be a risk for human infection [30]. Furthermore, factors such as changes in the management of cat faeces, changes in cat diet, delay in neutering, or increases in stray cats could limit the impact of the decreasing number of owned cats on human seroprevalence [31, 32]. This could explain the lack of decrease in *T. gondii* seroprevalence, despite a decreasing cat population. However, geographically looking at cats and *T. gondii* in the Netherlands, the percentage of households that own a cat is highest in the North and the highest absolute number of cats live in the West, and these regions also showed the highest seroprevalences. Therefore, there could possibly be a geographical link between the density of cats and infection with *T. gondii* in humans, even though direct cat contact may not be the most important risk factor for infection, for example through environmental exposure or consumption of locally produced food products.

Eating raw or undercooked meat has frequently been linked to the presence of antibodies to *T. gondii* [33, 34]. While this overall finding is relatively consistent between studies, the types of meat posing the greatest risk are variable. A systematic review of case-controlled studies published in 2018 found consumption of raw or undercooked beef and sheep meat, but not pork meat, to be important risk factors for *T. gondii* infection [35]. Other cross-sectional studies do find pork meat to be significantly associated with *T. gondii* seropositivity [36, 37].

In general, *T. gondii* infections seem to be most prevalent in sheep meat and least prevalent in beef and seem to differ for pork and chicken, with significantly higher prevalence rates in pork from organic farms compared with pork from conventional pig farming [35, 38]. Despite cattle having a high natural resistance to *T. gondii*, beef can contribute to human infection due to the higher likelihood of being consumed raw or undercooked compared with pork [34]. Therefore, it is difficult to speculate on the proportional contributions of different types of meat to human infection as dietary habits and environmental factors are constantly changing and differ between countries. In this study, ‘eating meat’ was a risk factor for *T. gondii* seropositivity, underlining the potential importance of eating meat as a major source of *T. gondii* infection even more. Confined husbandry and home freezing have likely contributed to a reduction in seroprevalence, but changes in dietary habits and the import of non-frozen meat could have counteracted this effect [39, 40]. Potential strategies to try to further reduce exposure to *T. gondii* include educating consumers, combined with improved labelling of the source of meat and the type of processing (i.e. farmed indoors or frozen), and measures to reduce infection in domestic animals (i.e. improved farm hygiene). There will always be trends that influence *T. gondii* infection pressure in different directions, and therefore, it is worthwhile to keep repeating serosurveys like the current one.

One of the strengths of this study is the application of an identical robust design with a nationwide representative sample for the third time with 10-year intervals, enabling the detection of changes in seroprevalence over the years and ensuring maximum comparability with previous studies [41]. In addition, we corrected selective responses using post-stratification weights.

A limitation of the design is that the measurement of seroprevalence is an indicator of *T. gondii*, but does not provide information on the time of infection. There could be a significant delay between infection and serological testing. Consequently, the results are conditional on the absence of any change in the respondents’ exposures over time. Lastly, the found association with the variables ‘eating meat’ and ‘eating raw or undercooked pork’ has to be interpreted with caution, as missing values within these variables were positively associated with seropositivity. The missingness of these variables was not related to any of the other variables and could be due to residual or unmeasured confounding.

## Conclusion

The decrease in *T. gondii* seroprevalence in the Netherlands between 1996/1997 and 2006/2007 stagnated in 2016/2017. Furthermore, 17.5% of women of reproductive age (15–49) were seropositive, leaving the majority of pregnant women susceptible to infection. The risk factor analysis underlines the importance of meat as a major source of *T. gondii* infection. However, it remains difficult to quantify exactly how the different risk factors relate to human infection, as environmental and socioeconomic factors can influence seroprevalences.

Better public awareness might reduce the risk of *T. gondii* and other infections, for instance sufficiently heating and freezing meat. Public health prevention measures may help further mitigate the risk of *T. gondii* infection for the Dutch population.

## Supporting information

van den Berg et al. supplementary material van den Berg et al. supplementary material

## Data Availability

The authors confirm that the data supporting the findings of this study are available within the article and its supplementary materials.

## Supplementary material

For supplementary material accompanying this paper visit http://doi.org/10.1017/S095026882300122X. click here to view supplementary material
